# Effects of maternal autoantibody exposure at the cellular level: implications for the developing brain

**DOI:** 10.3389/fpsyt.2026.1794161

**Published:** 2026-07-28

**Authors:** Katelien H. Blumenthal, Janna McLellan, Judy Van de Water

**Affiliations:** 1 Division of Rheumatology, Allergy, and Clinical Immunology, Department of Internal Medicine, University of California, Davis, Davis, CA, United States; 2 MIND Institute, University of California, Davis, Davis, CA, United States

**Keywords:** autism spectrum disorder, autoantibodies, collapsin response mediator protein, guanine deaminase, lactate dehydogenase, neuron-specific enolase (NSE), STIP1/HOP, YBOX-1

## Abstract

Maternal autoimmunity during pregnancy can have significant impacts on fetal development. In the case of maternal autoantibody-related autism (MARA), autoantibodies (aABs) reactive to fetal brain proteins in the circulation of pregnant mothers have been strongly associated with an offspring autism diagnosis. Clinical studies have identified the protein targets for the MARA aABs as well as maternal aAB patterns that are more commonly observed in mothers of children with autism, but not in mothers of neurotypical children. Additionally, rodent studies show that gestational exposure to MARA aABs alters offspring brain development and behavior. How MARA aABs cause changes at the cellular level is an area of active investigation. In this review, we discuss the potential mechanisms by which MARA aABs interact with key neuronal and non-neuronal cells as well as their target autoantigens in the developing brain. Additionally, we highlight the potential impacts of these interactions on the developmental trajectories of targeted cells, resulting in an autism diagnosis.

## Introduction

Autism Spectrum Disorder (ASD) is a behaviorally defined neurodevelopmental disorder with a heterogenous etiology (1). Maternal immune factors, including dysregulated cytokine profiles and presence of maternal autoantibodies (aABs) in the circulation of pregnant mothers, have been associated with altered fetal neurodevelopment and behavioral outcomes (2–7). In maternal autoantibody-related autism (MARA), specific patterns of maternal autoantibodies (aABs) in maternal circulation are associated with an autism diagnosis in offspring (8). MARA aABs have been characterized, determining that their target autoantigens are eight proteins that play important roles in neurodevelopment (9, 10). Previous clinical and preclinical rodent studies have elucidated the effects of MARA aABs on offspring gross brain development and behavior. However, few studies have determined the mechanisms by which MARA aABs interact with their target autoantigens and cells in the developing brain as well as their downstream cellular impacts. Herein, we discuss in detail the role of MARA aABs in autism, and how the developmental trajectories of cells in the fetal brain could be altered as a result of exposure to MARA aABs, leading to the development of MARA.

## Maternal antibodies and their role during pregnancy

During pregnancy, the maternal immune system plays a pivotal role in protecting both the mother and the fetus from infection. However, limitations in the inflammatory response to pathogens and other insults are essential to prevent harmful effects on the fetus (3). Changes in the maternal immune cell composition and modulation of the maternal response to infectious agents occurs to promote not only the health of both the mother and fetus but also to promote proper pregnancy progression (11, 12). In addition, the protection that the maternal immune system provides the fetus extends into the postnatal period, with early life antibody-mediated protection passed from mother to neonate via the placental transfer of immunoglobulin G (IgG) (13). The IgG subset of antibodies is unique in its ability to cross the placenta and gain access to fetal tissues. IgG transcytosis across the placenta is mediated by the neonatal Fc receptor (FcRn) expressed by syncytiotrophoblasts (14). By approximately 13 weeks of gestation, the human placenta begins functioning to supply nutrients and oxygen from the maternal compartment to the fetus (15). At this time maternal IgG is able to gain access to the fetus via the FcRn expressed on placental cells (13, 16). Mediating IgG placental transcytosis, syncytiotrophoblasts endocytose IgG, which binds endosomal FcRn at a low pH (17–19). FcRn binding IgG protects IgG from endosomal degradation (14, 20). Upon endosomal exocytosis into the fetal compartment, FcRn is exposed to a physiological pH, triggering the release of IgG into fetal circulation (14). As pregnancy progresses, the levels of maternal IgG crossing the fetal barrier increase, providing protection against agents against which the newborn would otherwise be defenseless (13). This mechanism has been studied to highlight the importance of maternal immunization as well as the potential harmful effects of maternal autoimmunity during pregnancy. The FcRn is not antigen-specific, meaning that it will indiscriminately bind IgG regardless of the antigen-specificity of each IgG. Harmful self-reactive autoantibodies (aABs) can therefore cross the placental barrier and gain access to the fetus.

Despite protective maternal immune responses, imbalances in maternal immune responses have been linked to altered fetal neurodevelopment. In the case of maternal immune activation (MIA), adverse maternal response to infections, autoimmune disorders, allergy, asthma, and obesity, have been linked to increased risk for offspring neurodevelopmental disorders such as ASD, attention deficit hyperactivity disorder (ADHD), and schizophrenia (4, 21–27). For example, animal models have been used to show that viral stimulant poly(I:C) (i.e. infectious agents) resulted in dysregulated cytokine levels, such as interleukin (IL)-17A and IL-6, and were linked to aberrant offspring neurodevelopment and behavior (6, 7). While maternal immune cell and cytokine responses have been linked to altered fetal neurodevelopment in MIA, transfer of pathogen-specific and autoreactive antibodies has also been linked to adverse developmental outcomes. Fetal pathologies have been associated with several IgG-linked maternal autoimmune conditions, including fetal congenital heart block in newborns born to mothers with primary systemic lupus erythematosus (SLE) (28) and transient fetal myasthenia gravis in newborns born to mothers who express anti-acetylcholine antibodies (29). In addition, mothers with SLE expressing aABs reactive to N-methyl-D-aspartate receptor (NMDAR) had offspring with altered neurodevelopmental outcomes (30) which was recapitulated in a mouse model and shown to lead to disrupted cortical development and aberrant cognitive functioning and reflexes (31). Further, IgG targeting human cytomegalovirus and mycobacterium antigens are linked to an increased ADHD diagnosis in offspring (32). Moreover, altered neurodevelopment has been observed in children born to mothers who express aABs that bind to proteins in the fetal brain (5, 9, 33–35). Downstream studies have further described the association between anti-brain aABs and an autism diagnosis in offspring, elucidating a novel subtype of autism: maternal autoantibody-related autism (MARA).

## Maternal autoantibody-related autism

In the subtype of autism known as MARA, mothers produce characteristic patterns of aABs that are highly specific to an autism diagnosis. Early clinical studies first identified a subset of mothers of autistic children who produced aABs reactive to fetal brain proteins at 37, 39, and 73 kDa (33). This reactivity was not observed in mothers of neurotypical children. Follow-up clinical research identified the MARA antigens to which the aABs were binding included lactate dehydrogenase A and B (LDHA/B), guanine deaminase (GDA), stress-induced phosphoprotein 1 (STIP1), collapsin response mediator proteins 1 and 2 (CRMP1/2), and Y-box-binding protein 1 (YBOX-1) (9). Further studies later identified an eighth antigen, neuron-specific enolase (NSE) (10). Following identification of the target proteins, it was determined that maternal reactivity to specific combinations, or patterns, of the MARA antigens predicted ASD diagnosis. Patterns include CRMP1+CRMP2, CRMP1+GDA, CRMP2+STIP1, CRMP1+STIP1, GDA+YBOX-1, STIP1+NSE, LDHA+YBOX-1, LDHB+YBOX-1, and LDHA/B+CRMP1+STIP1 which were observed in mothers of children with autism, but rarely in mothers of neurotypical children (8, 36, 37). The initial study identified maternal aAB patterns in plasma of 10-18% of California mothers with children with autism enrolled in the Childhood Autism Risks from Genetics and the Environment (CHARGE) study (8). A subsequent study that assessed MARA patterns using plasma from pregnant mothers in Southern California enrolled in the Early Markers for Autism (EMA) study found similar maternal aAB predictivity of offspring autism diagnosis (10% of mothers) (37). To increase the geographical heterogeneity of study populations, clinical cohorts from Arkansas and Philadelphia were assessed for MARA prevalence and found incidence of MARA patterns in 26% and 21% of mothers, respectively (36).

## Understanding the MARA pathology

Longitudinal magnetic resonance imaging (MRI) studies identified discrepancies in brain volume between autistic and neurotypical children (38), consistent with the observed early brain overgrowth in autistic children (39). To assess the unique impact of MARA IgG on offspring neurodevelopment, MRI studies investigated brain volume differences across MARA, other autistic, and neurotypical children (40). MARA children exhibited both abnormal gross and regional brain enlargements with respect to neurotypical and other autistic children (40). This study indicates a MARA-specific pathological impact on child brain development when compared to neurotypical and other autistic individuals.

Several iterations of preclinical rodent models have been utilized to assess how gestational exposure to the MARA aABs impacts offspring neurodevelopment and behavior. Mouse and rat models have shown that gestational exposure to the LDHA/B+CRMP1+STIP1 pattern of aABs results in behavioral changes related to autism, such as increased self-grooming behavior and a reduction in social interaction and play behaviors (41–44). Neuroanatomically, it was also found that offspring exposed to MARA aABs had differential total brain volume, a phenotype often observed in clinical autism cases (40, 42, 43). These studies observed sex-based differences in the impacts of MARA aABs on offspring brain volume. In mice, enlargements were primarily observed in MARA female brains, with enlargements in cerebral cortex, white matter, cerebral nuclei, and cerebellum (43). In rats, males had decreased total brain volume, but increased volume in the somatosensory cortex, while females had increased total brain volume driven by increased volume in most brain regions (42).

Studies using rodents exposed to LDHA/B+CRMP1+STIP1 aABs during pregnancy found that IgG localized with neural progenitor cells (NPCs) in the embryonic mouse brain and neurons in the early postnatal rat brain (42, 44). Additionally, the aABs impacted murine NPC development, inducing premature NPC migration and increased proliferation in the embryonic offspring brain, and neuron morphology, leading to increased soma size and decreased dendritic spines in the adult offspring brain (44, 45). Expanded work using rats prenatally exposed to LDHA/B+CRMP1+STIP1 aABs well as the clinically relevant patterns CRMP1+CRMP2, CRMP1+GDA, and STIP1+NSE noted additional changes in the levels of immune signaling molecules and differential gene expression in the early postnatal brains of offspring (46). Lower levels of several cytokines important for early brain development were observed in the MARA aAB-exposed offspring when compared to saline-exposed controls, including interleukin (IL)- 2, IL-1β, and interferon gamma (IFNγ). Lower levels of vascular endothelial growth factor (VEGF), which is important for angiogenesis in the brain, were also observed in the brains of CRMP1+CRMP2-exposed offspring when compared to CRMP1+GDA-exposed offspring. Upregulation of *wnt* signaling genes and of genes that activate the mitogen-activated protein kinase (MAPK) cascade and downregulation of NFκB signaling genes were also observed (46). Dysregulation of genes involved in these signaling pathways have been implicated in clinical autism (47, 48), suggesting that gestational exposure to MARA aABs can affect the genes involved in these signaling pathways, although the underlying mechanism of this impact remains unknown.

## Model limitations and gaps in knowledge

Multiple studies have assessed the association of MARA aAB patterns and a clinical offspring autism diagnosis (8, 36, 37). However, there are some limitations to current work with MARA. Presence of a single maternal aAB alone does not predict risk of autism, but instead, the maternal aABs must occur in specific patterns of two or more aABs to predict risk of autism (8). It is currently unclear why specific aAB patterns cause autism, and not just maternal aABs in isolation, as well as the etiology of these aABs. Early studies on MARA indicated that occurrence of maternal aABs with reactivity to proteins of 37 and 73 kDa, were not linked to any specific autoimmune conditions (5). Another study showed expression of aABs specifically reactive to proteins at 37 and 73 kDa occurs in mothers with the *MET* C allele (49). However, this allele was highly associated with lower IL-10 expression, and therefore, lowered immunoregulatory function (49). Thus, the allele is not necessarily associated with direct aAB production. Moreover, previous clinical studies looked at the presence of maternal aAB patterns in four clinical cohorts, and while overall population incidence was similar across studies (10-26% of autism cases), predominance of each individual pattern varied between study populations (8, 36, 37). More specifically, the most prevalent pattern in the Northern California CHARGE study was the CRMP1+GDA pattern, followed by the CRMP1+CRMP2, STIP1+NSE, and CRMP2+STIP1 patterns (8). Conversely, the most significant patterns predictive of autism risk in the Southern California EMA study were CRMP1+CRMP2, CRMP2+STIP1, CRMP1+STIP1, LDHA+YBOX, and GDA+YBOX, while the patterns STIP1+NSE and CRMP1+GDA did not significantly predict autism risk with respect to intellectual disability and neurotypical controls (37). While another study was used to test MARA pattern predictivity of autism in other geographically distinct study populations, the study cohorts were small and therefore could not be used to evaluate predictivity of individual MARA patterns (36). Overall, while MARA aAB patterns can be used to predict ASD risk clinically, the prevalence of specific patterns and their predictivity of ASD is variable across study cohorts.

ASD is a behaviorally defined neurodevelopmental human disorder; however, many studies use rodent models to better understand the neurobiological and behavioral outcomes. Rodents have many advantages in studying neurodevelopmental disorders, including genetic and other biological manipulations, short gestational periods, high litter counts, and ability to access the brain tissue for biological and functional measurements (50). Additionally, rodent behavioral tests have been developed to map to the developmental behaviors of humans from birth to adulthood (50–52). Such behavioral tests can be used to characterize autistic-like behaviors in rodents used to model ASD (51, 52). However, despite these advantages, rodent models pose a few limitations including smaller and less complex tissues, less complex behaviors than humans, and shorter duration of pregnancy and brain development which does not directly map to human gestational and brain developmental timepoints (50, 51). In addition, the etiology of autism is heterogenous and does not stem from a sole genetic or environmental cause, and thus, can be induced in a variety of ways (51). Thus, studies evaluating the symptomology and severity of ASD in rodent models generally results in varied biologic and behavioral outcomes (51).

Rodent studies have evaluated the impact of MARA aABs on altered offspring brain structure and molecular profiles, and autism-like behaviors, including social play, communication, and repetitive and stereotyped behaviors (41–44, 46). Still, the direct mechanism of interaction between MARA aABs and their target proteins, which are primarily intracellular, are unknown. Further, the artificial induction of MARA aAB production is variable, and does not provide insight into the level of aABs required to induce pathology. The effect of MARA aABs on individual populations of neuronal and non-neuronal cells in the brain have not been extensively characterized in rodent or human models. While the exact mechanisms of interaction between the MARA aABs and their target proteins and cells in the developing brain remain unclear, preclinical findings suggest neurodevelopmental changes at the cellular level. More specifically, in MARA rodent studies, IgG was found to colocalize with NPCs in mouse brains intracerebroventricularly (ICV) injected with MARA IgG as well as with neurons in rat brains passively exposed to aABs during pregnancy (42, 44). Embryonic day (ED) 14.5 embryos ICV injected with MARA IgG exhibited altered embryonic NPC migration and proliferation as well as increased adult neuron soma size and decreased dendritic spine numbers (44, 45). These findings indicate an impact of MARA aABs on early and late neuronal profiles; however, ICV injection artificially exposes offspring to MARA aABs. Thus, there is a gap in knowledge as to how passive transfer of MARA aABs across the placenta during pregnancy impacts individual cell populations. Maternal-fetal IgG transfer studies show that maternal IgG begins to enter the fetal compartment as early as embryonic week (EW) 13 in humans (16) and ED12.5 in rodents (53) which is prior to complete blood-brain barrier (BBB) closure around EW14 in humans (54, 55) and ED15.5 in rodents (56). Thus, MARA aABs can gain access to the developing brain while the BBB is still permissive (53). During this gestational timeframe, the brain is composed of cells, including progenitor cells (neural and oligodendrocyte), neurons, microglia, endothelial cells, and pericytes (57). Each of these cells plays a role in the formation and function of the developing brain.

Future studies should aim to address how MARA aABs interact with cells and their target antigens in the developing brain, and to determine the mechanism of those interactions. Additionally, since ASD is a human-defined behavioral disorder, understanding the cellular impacts of MARA aABs on rodent cells will allow for the translation of cellular MARA pathology to human cerebral organoid models. Herein, to address the potential mechanisms and impact of MARA aABs on cells in the developing brain, we review the major neuronal, glial, and non-neuronal cell types in the developing brain and their expression of MARA autoantigens, as well as postulate the impacts of MARA aAB exposure on each cell type.

## NPCs and neurons

Neural progenitor cells (NPCs) are multipotent cells important for cortical development (58). A subset of NPCs, termed radial glial cells (RGCs), primarily undergo asymmetric divisions at the ventricular zone (VZ) yielding an identical daughter progeny and an intermediate progenitor cell (IPC) (58, 59). IPCs subsequently migrate to the subventricular zone (SVZ) where they symmetrically divide into two daughter neurons (58, 59). Terminally differentiated neurons migrate to the cortical plate, forming the cerebral cortex (58). During late pregnancy, RGCs will also differentiate into astrocytes (discussed in the next section) (60, 61). Ultimately, temporal and spatial regulation of divisions and migration of NPCs and neurons are critical for proper neuronal and overall cortical development. Alterations to NPC divisions and NPC and neuron migration have been linked to aberrant neurodevelopment (58). For example, mutations in the *Fragile X Mental Retardation 1* (*FMR1*) gene observed in Fragile-X Syndrome and ASD have been found to lead to increased IPC production and depleted levels of RGCs, as well as delayed transition of neurons into a bipolar morphology, which can be linked to subsequent irregular neuronal migration and impaired neuronal networks (62, 63). Thus, abnormal NPC and neuron structure and function in the developing cortex likely contributes to the pathogenesis of neurodevelopmental disorders.

Early preclinical MARA mouse studies showed that following ICV injection with LDHA/B+CRMP1+STIP1 MARA IgG offspring RGCs prematurely migrated from the VZ to SVZ and had altered proliferation at the SVZ (44). In rodent brains, RGC migration from the VZ to the SVZ occurs during late neurogenesis and early astrogenesis (59), which occurred in control but not MARA IgG-exposed offspring brains (44). In addition, this exposure to MARA IgG resulted in decreased dendritic spine density and increased soma size of neurons in adult mice cortices, indicating altered cortical structure and neuronal morphology and function due to MARA aAB exposure (44, 45). More recently, a preclinical MARA rat model was used to show that gestational exposure to maternal aABs results in the deposition of IgG adjacent to neurons in the early postnatal brain (42). The mechanism of interaction between IgG and neurons is unknown. However, neurons are a likely target for MARA aABs as they possess various potential of IgG internalization, critical for binding intracellular target autoantigens, including clathrin-mediated endocytosis and synaptic uptake (64–66). In addition, neurons express Fc receptors (FcRs), which can bind IgG in the developing brain, triggering clathrin-mediated endocytosis (67–70).

**Figure 1:**
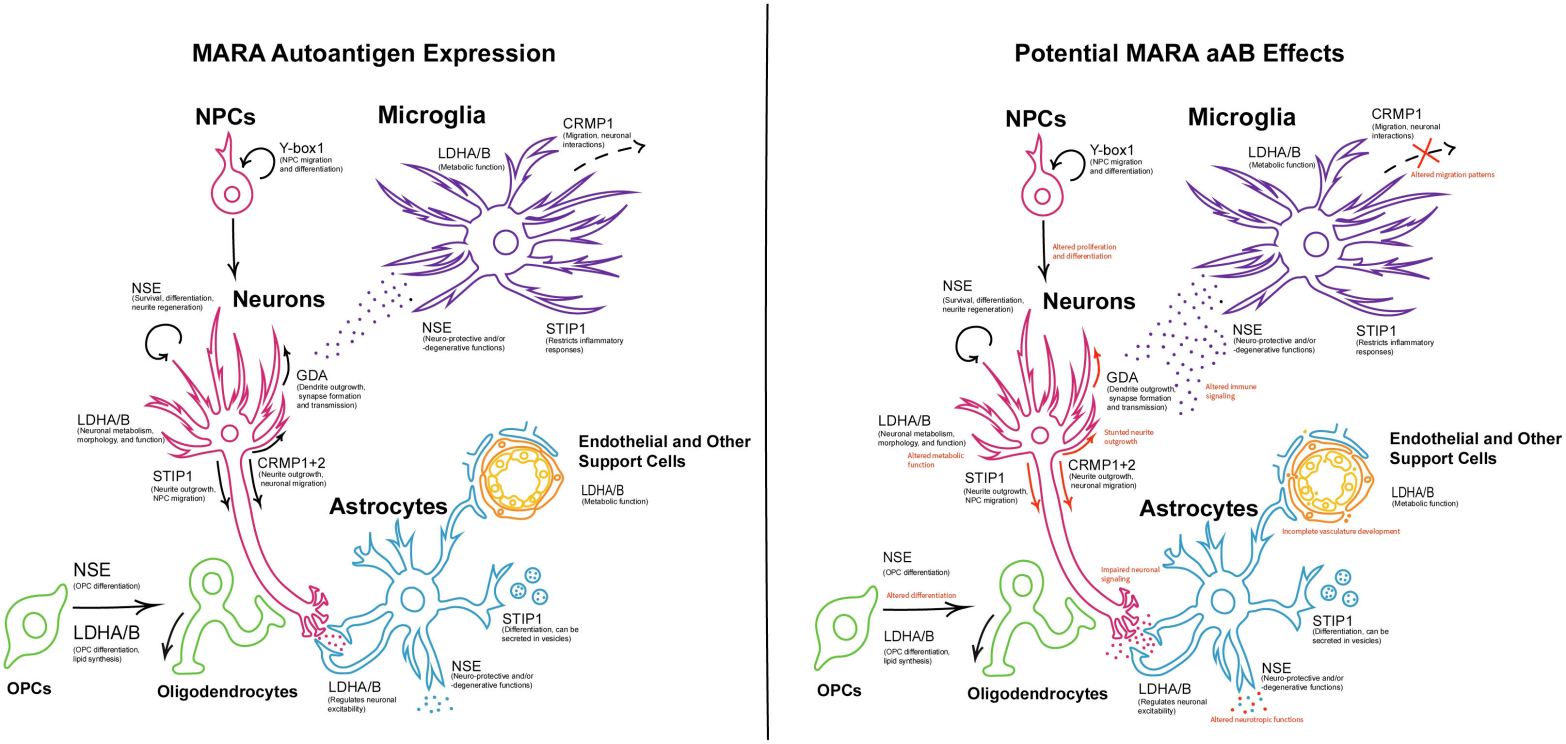
Cellular expression of MARA autoantigens in the developing brain and the potential pathogenic effects of MARA aAB exposure. Neuronal and non-neuronal cells interact with one another to drive neurotypical brain development. These cells, including neurons, astrocytes, microglia, oligodendrocytes, and endothelial cells, express MARA autoantigens and are therefore susceptible to MARA aABs. While the cellular effects and mechanisms of MARA aABs are not fully understood, we hypothesize that MARA aAB exposure can impact cellular neurodevelopment of various neuronal and non-neuronal cells in the developing brain via intercellular interactions. The potential developmental impacts of MARA aAB exposure are highlighted here (red) and include aberrant neuronal signaling and connectivity, altered progenitor and glial cell migration, incomplete vasculature formation, and abnormal immune signaling in the brain.

Neurons express all identified MARA autoantigens, including CRMP1, CRMP2, STIP1, GDA, NSE, YBOX-1, and LDH (**Figure 1**). The primary role of CRMP1 and CRMP2 is to regulate axonal growth cone collapse, neurite outgrowth, dendrite development, and neuronal migration (71–75). Moreover, a previous study showed that an individual with a neurodevelopmental disorder expressed a *de novo* variant of CRMP1, implicating the role of CRMP1 in proper neurodevelopment (76). In addition, various rodent models with altered CRMP1 and CRMP2 expression exhibit aberrant neuronal migration, neurotransmission, and dysregulated dendrite outgrowth, resulting in impaired dendritic spine and synapse formation (71, 73–75, 77). STIP1 is a chaperone protein that interacts with heat shock protein (HSP) 70 and HSP90 (78). STIP1 plays important roles in neuroblast migration to the SVZ and maintenance and promotes neuronal neurite formation and neuroprotection (78, 79). STIP1 is critical for embryonic development, as its deletion is lethal to early embryonic mice (78). Previous MARA studies showed that administration of IgG, including anti-STIP1, resulted in altered NPC migration to the SVZ, similar to neuroblasts in previous STIP1 blocking studies (44, 79). GDA plays an important role in neuronal dendrite formation (80), synaptogenesis (81), and electrical activity (82). Interruptions to neuronal GDA activity may alter neuronal activity and networks. Depending on the injury, disease, and microenvironment, NSE controls neuronal survival, differentiation, and neurite regeneration via activation of phosphatidylinositol-4,5-bisphosphate 3-kinase (PI3K) and mitogen-activated protein kinase (MAPK) signaling pathways (83). Dysregulation of these pathways have been linked to clinical autism (47, 84) and have been found in the brains of preclinical MARA rats (46), and therefore may be targets for MARA pathogenesis. Studies of YBOX-1 in early brain development indicate that this cytosolic protein is critical for NSC proliferation and differentiation, and loss of YBOX-1 is lethal to embryonic mice (85, 86). MARA aABs targeting YBOX-1 during neurodevelopment may prevent neurotypical NPC development. Finally, LDHA/B knockout models indicate that the metabolic enzymes play an important role in maintaining neuronal metabolism, morphology, and functions important for cognition, memory, and emotional behaviors (87, 88).

Altered NPC/neuron development are largely implicated in previous MARA rodent studies (42, 44, 45). Ultimately, the expression of MARA autoantigens and pathways for antibody internalization indicate neurons are a probable target for the pathological activity of MARA aABs.

## Astrocytes

Differentiated astrocytes arise during late embryonic and early postnatal brain development (60, 61). Initially, RGCs undergo massive proliferation and differentiation into IPCs and neurons. In later stages of embryonic development, RGCs begin expressing astrocyte lineage-specific markers, driving their postnatal maturation into astrocytes (60, 61). Thus, proper RGC development is important for downstream astrogenesis. Astrocytes are primarily responsible for formation and regulation of the blood brain barrier, maintenance of synapses, neuronal development, and production and integration of diverse neuromodulatory and immune signals (89, 90). More specifically, astrocytes regulate neuronal excitability by reuptake and processing of neurotransmitters, such as glutamate, and regulation of extracellular ion levels (89). In addition, astrocytes regulate neuronal synapse birth, maintenance, transmission, and removal by producing factors critical for each of these processes (89). Aside from roles in neuronal maintenance, astrocytes produce factors which are integrated by pericytes and endothelial cells to maintain the BBB, regulating permeability of this barrier system (91). Altered astrocyte profiles have been observed in neurodevelopmental and psychiatric disorders (60). For example, astrocyte-specific deficiencies in Fragile-X Syndrome models have been linked to delayed neuronal dendrite maturation and excitatory synapse development and increased neuronal excitability, linked to dysregulated glutamate reuptake (92, 93). This underscores the important roles that astrocytes play in neuron development, and thus, the impact potential astrocyte dysregulation can have on neuron maturation and function.

While the cellular targets and mechanisms of MARA IgG are unknown, astrocytes exhibit features that indicate potential vulnerability to MARA aABs. Astrocytes express a multitude of surface proteins, including FcRs, which are a potential route by which MARA IgG can be internalized and bind their target autoantigens (67, 68). MARA autoantigens, including STIP1, NSE, and LDH, are expressed by astrocytes and play important roles in astrocyte development (**Figure 1**). STIP1 is expressed by astrocytes in which they promote differentiation via MAPK signaling, and survival via protein kinase A (PKA) signaling (78). Differential expression of genes involved in the MAPK signaling cascade was observed in MARA offspring rat brains (46), indicating downstream impact of MARA targeted aABs. To promote neuronal survival and differentiation, astrocytes also secrete extracellular vesicles containing STIP1, providing a potential mechanism through which MARA IgG can bind their target antigen and enter cells (94, 95). NSE expression has been found in reactive astrocytes and therefore may be linked to the neuro-protective and/or -degenerative functions of astrocytes (83). Astrocytes also express LDHA, which regulates neuronal excitability and social behaviors of rodents (96). Reduction of astrocyte LDHA expression deteriorated neuronal firing and increased social avoidance behaviors, indicating the importance of regulated astrocytic LDHA expression (96). Overall, targeting astrocytic MARA autoantigens may be linked to observed transcriptional and neuronal changes in MARA offspring brains (44–46).

## Microglia

Microglia are commonly known as the resident immune cells of the brain, where they function to support neurons and provide immunological defense and surveillance (97). Microglial progenitors derive from the fetal yolk sac and migrate to the developing nervous system. Cortical infiltration by microglial cells begins as early as the fourth week of gestation in humans and embryonic day ED11.5 in the mouse and ED13 in the rat, undergoing distinct patterns of migration throughout the fetal brain as they establish (54, 98, 99). Although they make up a small portion of the total number of brain cells, differentiated populations of microglia can be detected in the human fetal brain at around 35 weeks of gestation (100). Both the maturation and differentiation of microglial populations continue until birth in humans and as late as postnatal day 14 in rodents (101). Once within the brain, microglial populations are maintained through the expansion and differentiation of the early colonizing cells with little contribution from myeloid progenitors in the periphery (102). Through the course of fetal and postnatal brain development, they play key roles in the development of other cells within the brain. By ED16 microglia preferentially reside near the VZ and SVZ, where they assist in the regulation of neurogenesis and oligodendogenesis, controlling the numbers of progenitor cells in the developing cortex (103–105). During neurodevelopment, microglia produce immune analytes that regulate cell development, activation, and migration, such as IL-1β, IFNγ, IL-6, and TNFα. Previous studies indicate that microglial production of cytokines IL-1β and IFNγ promotes neurogenesis while IL-1β and IL-6 promote oligodendrogenesis (106). An imbalance in the production of immune analytes has been linked to autism and shown to inhibit neurogenesis and oligodendrogenesis in the SVZ of the early postnatal rat brain (97, 106). Additionally, microglia phagocytose NPCs within the developing brain, regulating the number of neuronal cells present in the CNS, and ultimately the structure and function of the cortex (99). It has been shown that overactivation of microglia via LPS exposure in the context of MIA (driven by immune analyte changes), leads to reduction in NPC and mature neuron production, while suppressing microglial activation leads to increases in NPCs and mature neurons, without changes to NPC apoptosis (99). Thus, immune-mediated dysregulation of microglia can be linked to increased brain size and cortical neuronal numbers observed in individuals with ASD (38, 107). Moreover, later in neuronal development, in a process termed “synaptic pruning”, microglia phagocytose, or “prune”, neuronal synapses within the developing brain, refining neuronal functional connections (105). The interface between microglia and neurons in synaptic pruning is largely driven by microglia surface expression of chemokine signaling receptor CX3CR1, which responds to the chemoattractant CX3CL1 produced by neurons (108). Synaptic pruning is initiated via microglial production of complement protein C1q, which triggers the complement cascade, ultimately tagging synapses/cells for phagocytosis (109). This process is critical for neuronal connectivity and signaling as it refines the number and types of neuronal connections. Synaptic activity is also regulated by microglial cytokines IL-1β, TNF, and IL-6 which act on receptors at the synapses to control excitatory transmission (110). Dysregulation of these cytokines have been observed in rodent models of neurological and psychiatric disorders (110). For example, microglia-produced TNF is linked to synaptic potentiation and resulting anxiety-like behaviors (111). In preclinical MARA studies, the early postnatal MARA rat offspring brains exhibited altered production of microglia-produced cytokines, including IL-1β (46). These same brains had altered gene expression related to neuronal differentiation and synaptic potentiation (46). Together, these findings indicate a potential role of microglia and their immune byproducts in MARA pathology.

Similar to astrocytes, microglial cells express FcRs, which allows for interaction with IgG (68). Thus, when exposed to auto-reactive IgG, microglia are susceptible to autoimmune-mediated damage via FcR interaction and uptake–a potential mechanism for MARA IgG internalization. Rodent studies have found that microglia express several of the MARA aAB targets, including CRMP1, STIP1, NSE, and LDHA/B (83, 112–114) (**Figure 1**). The expression of these MARA aAB targets combined with the ability to interact with IgG suggests their vulnerability to MARA aABs during development. Of the MARA aAB targets, LDHA/B is of interest due to the role of lactate metabolism in early brain development (115). Due to the high energy requirements needed by microglia, an inability to perform the glycolytic functions mediated by LDHA/B would be detrimental to their function. While studies addressing the role of CRMP1 in developing microglia are lacking, alterations in the function of CRMP1 could inhibit their ability to properly migrate and interact with developing neurons (71, 116).

The early development of microglia and their colonization of the fetal brain represent a highly vulnerable period. It is hypothesized that due to the role of microglia in neuron and oligodendrocyte development during embryonic and early postnatal periods, potential changes to microglial function due to aAB exposure at these critical periods could potentially explain why behavioral and neuroanatomical changes in adult brains (MARA rodent models) are observed.

## OPCs and oligodendrocytes

Oligodendrocyte progenitor cells (OPCs), precursor cells to oligodendrocytes, arise as early as ED11.5 in the cerebellum and ED12.5 in the forebrain in rodents (117). OPCs in the early forebrain are derived from the SVZ; these cells migrate throughout the developing cortex and undergo proliferation to maintain OPC levels followed by downstream differentiation into oligodendrocytes (117). OPC migration, self-renewal, and maturation into oligodendrocytes are all important for angiogenesis, blood-brain barrier maintenance, neuronal connectivity, and downstream axonal myelination (117, 118). Altered oligodendrocyte development has been linked to autism-like behaviors in rodents (119, 120). More specifically, rodents with deficits in myelin production and oligodendrocyte maturation exhibited ASD-like behaviors (119, 120).

A previous MARA rodent study showed gestational exposure to MARA aABs resulted in increased white matter tracts volume in offspring brains (43). Such differences could in part be linked to altered OPC/oligodendrocyte populations and myelin production. OPCs and oligodendrocytes express FcRs, specifically FcγR, which can bind IgG present in the brain (68). OPCs/oligodendrocytes express various MARA proteins including NSE and LDH (121, 122) (**Figure 1**). NSE is expressed by differentiating OPCs, as it plays a role in differentiation into oligodendrocytes (121). LDH is utilized by oligodendrocytes for lipid synthesis and promotes OPC differentiation (122). Alterations to NSE and/or LDH may result in altered oligodendrocyte maturation which could manifest in white matter alterations previously observed in MARA rodent models (43).

## Endothelial and supportive cells

Outside of glial cells, the central nervous system is also comprised of other cell types that help support and protect the brain including specialized endothelial cells and pericytes. Within the brain, endothelial cells and pericytes work alongside neurons, astrocytes, and microglia to form the neurovascular unit (NVU) and ultimately help to form a functioning blood brain barrier (BBB) (123). Brain microvascular endothelial cells (BMECs) also have roles promoting the migration of neurons and accelerating neurite outgrowth. Disruption of and altered development of BMECs have been linked to neurological disorders such as schizophrenia (124).

Angioblasts, the progenitors of BMECs, are recruited to the developing nervous system following the closure of the neural tube, as early as ED8.5 in the mouse (125). Angioblasts surround the neural tube to generate a primitive vascular network that becomes more ramified as gestation progresses. This process is regulated primarily by the vascular endothelial growth factor A (VEGF-A) (126, 127). During gestation, VEGF-A is primarily produced by the placenta, more specifically by syncytiotrophoblasts and the invasive chorionic trophoblasts, the uterine endometrium, and the corpus luteum. This promotes angiogenesis, vascular permeability, and placenta development throughout gestation (128, 129). In the fetal space, VEGF-A is crucial for fetal brain development, where it regulates neurogenesis, angiogenesis, and vascular permeability. It acts as a signaling protein that stimulates new blood vessel growth, with high expression during critical developmental periods from gestational weeks 9–40 in humans. Production of VEGF-A in the developing fetal brain has been detected the subependymal germinal layer, the choroid plexus, and the cerebral cortex, where expression peaks between 20–28 weeks (130). Thus, throughout neurodevelopment, the surrounding vasculature penetrates into the developing brain to aid in neurogenesis (131). Interestingly, in the MARA rat model, it was observed that VEGF was significantly lower in the brains of MARA aAB-exposed offspring at postnatal day 2 (46). While BMECs do not express significant amounts of many of the MARA protein targets, the fact that MARA aAB exposure leads to a decrease in VEGF, a key growth factor for BMECs, suggests the potential for a downstream effect of MARA aABs on these critical cells (**Figure 1**). For example, if MARA aABs directly interact with neurons, dysregulating their production of VEGF, BMEC organization into vasculature and function in the developing brain may be altered. This highlights the idea that MARA aABs may not only have direct impacts on the cells they interact with but also indirect, downstream impacts on adjacent cells in the developing brain. Further, there is evidence that these specialized endothelial cells help regulate neurogenesis through the secretion and maintenance of lactate (132). Altered function of the LDHA/B proteins due to MARA-AB exposure, could thus impact this key function and alter downstream cellular development and function (**Figure 1**). Following a similar timeline, pericytes can be detected in the embryonic mouse brain at approximately ED10 (133). Pericytes also rely on lactate metabolism for energy production, thus impacts on LDHA/B expression could subsequently alter their function and impact long-term BBB homeostasis (134).

## Autoantibody internalization and interaction with intracellular proteins in neurological disorders: potential mechanisms of cellular interaction

MARA autoantigens are expressed by various cells in the developing brain, yet studies have not determined the mechanism of interaction between MARA aABs and cells that express their target autoantigens. Except for STIP1, MARA autoantigens are primarily intracellular, and thus, direct targeting of these autoantigens must occur via aAB internalization. While the mechanism(s) of MARA aABs are unknown, the etiology and pathology of multiple other neurological disorders have been linked to the internalization and action of aABs targeted at intracellular autoantigens. Thus, to highlight potential routes of MARA aAB action, we have reviewed various mechanisms of IgG internalization implicated in neurological dysregulation, including clathrin-mediated endocytosis and endocytosis into synaptic vesicles, resulting in aberrant cellular function and cell death (64, 65). More specifically, aABs targeting intracellular heterogeneous nuclear ribonucleoprotein A1 (hnRNP A1) have been linked to multiple sclerosis (MS) and human T-lymphotropic virus type-1 associated myelopathy/tropical spastic paraparesis (HAM/TSP) diagnoses (64, 66). A previous study showed that neurons internalized anti-hnRNP A1 IgG isolated from MS and HAM/TSP patients following exposure *in vitro* (64, 66). In this model, aABs were internalized via clathrin-mediated endocytosis and targeted intracellular hnRNP A1 in the cytoplasm of neurons, leading to increased neuronal apoptosis and ATP depletion (64, 66). Moreover, autoimmune retinopathy has been linked to expression of anti-alpha-enolase aABs which were found to be internalized by retinal cells (135). Retinal cells that internalized anti-alpha-enolase were found to inhibit enolase function and promote glycolytic dysregulation, ultimately leading to cell death (135). Finally, stiff person syndrome has been linked to the epitope-specific internalization of aABs targeted against neurotransmitter amphiphysin into synaptic vesicles (65). Anti-amphiphysin antibody internalization results in reduced synaptic function (65). Thus, a similar pathogenic mechanism could be envisioned for MARA aABs.

## Discussion

Clinically, MARA has been identified as a subtype of autism in which specific patterns of maternal aABs are linked to an autism diagnosis in offspring (8). Early studies identified the target autoantigens (9, 10) as well as the linkage between maternal aABs and aberrant offspring behaviors (33). Longitudinal imaging studies showed that these aABs are linked to aberrant phenotypes of brain volume of patients with autism (40). Rodent and non-human primate studies have shown that gestational exposure to MARA aABs can lead to aberrant offspring behaviors (41–44, 136), brain regional volumetric differences (42–44, 136), lower neuronal dendritic spine numbers (45), increased neuronal soma size (44), and increased proliferation of progenitor cells in the SVZ (44). Despite these findings in multiple model systems, there are still many unanswered questions about MARA. First, it is unclear which cell population(s) are directly targeted by MARA aABs due to the multicellular expression of the autoantigens throughout the brain. Additionally, it is unclear if and how MARA aABs interact with cells and their target autoantigens in the developing brain. Finally, findings on the downstream impact of these aABs on cellular function and how that relates to observed behaviors are limited to rodent models. Ongoing work utilizes both *in vitro* and *in vivo* MARA model systems to address these questions. To determine which cells are directly targeted by MARA aABs, current studies are looking at the deposition of IgG with respect to MARA autoantigens and cells in the developing rat brain. To bridge the gap between rodent and human brain development, ongoing work is using cerebral organoid exposed to MARA aABs to determine the single cell impacts of these aABs on gene expression and cellular function in human brain cells. Additional MARA studies can use this organoid system to further elucidate the mechanisms by which antibodies interact with neuronal and non-neuronal cells as well as intracellular autoantigens.

MARA aABs can be used as biomarkers for early detection of autism in offspring, allowing parents and children to obtain appropriate resources. Various therapeutics have been used to treat antibody-mediated autoimmune disorders during pregnancy (137, 138). Intravenous immunoglobulin (IVIG) or plasmapheresis are potential therapeutic methods that can be used in pregnant mothers with MARA; they have both been used to restrict the effects of autoimmune disorders such as myasthenia gravis (138, 139). Both IVIG and plasmapheresis function to “dilute” the effects of pathogenic aABs via the administration of innocuous IgG (IVIG) or the specific removal of pathogenic aABs (plasmapheresis). FcRn blocking therapies are another potential therapeutic, blocking the transfer of IgG from maternal to fetal circulation and reducing half-life of IgG (140). FcRn blocking therapies have been used in the treatment of IgG-mediated gestational autoimmune disorders such as autoimmune hemolytic anemia (140). However, recent studies have indicated a beneficial role of innocuous maternal IgG in offspring brain development (141). Thus, engineering novel antigen-specific FcRn-targeted immunotherapies may be critical to specifically target pathogenic aABs for degradation without loss of non-pathogenic IgG, as was previously done with myelin oligodendrocyte glycoprotein (MOG)-specific aABs (142). In the case of MARA, it is critical that future studies examine the effects of multiple therapeutic approaches.

Overall, neuronal, glial, and other non-neuronal cells in the developing brain utilize the proteins targeted by MARA aABs throughout neurodevelopment. Understanding how these pathogenic aABs target their autoantigens as well as the cells that these aABs target in the central nervous system will allow us to elucidate the pathogenetic mechanisms of MARA.
